# A multi-target antisense approach against PDE4 and PDE7 reduces smoke-induced lung inflammation in mice

**DOI:** 10.1186/1465-9921-10-39

**Published:** 2009-05-20

**Authors:** Marylène Fortin, Hélène D'Anjou, Marie-Ève Higgins, Jasmine Gougeon, Paméla Aubé, Kamel Moktefi, Sonia Mouissi, Serge Séguin, Rosanne Séguin, Paolo M Renzi, Luc Paquet, Nicolay Ferrari

**Affiliations:** 1 Topigen Pharmaceuticals Inc, 2901 Rachel Street East, Room 13, Montreal, Quebec, H1W 4A4, Canada; 2 CHUM Research Center, Notre-Dame Hospital, 2065 Alexandre de Sève, Room Z-8905, Montreal, Quebec, H2L 2W5, Canada; 3 Institut de Pharmacologie, Université de Sherbrooke, 3001 12e Avenue Nord, Sherbrooke, Quebec, J1H 5N4, Canada

## Abstract

**Background:**

Recent development in the field of COPD has focused on strategies aimed at reducing the underlying inflammation through selective inhibition of the phosphodiesterase type IV (PDE4) isoform. Although the anti-inflammatory and bronchodilator activity of selective PDE4 inhibitors has been well documented, their low therapeutic ratio and dose-dependent systemic side effects have limited their clinical utility. This study examined the effect of 2'-deoxy-2'-Fluoro-β-D-Arabinonucleic Acid (FANA)-containing antisense oligonucleotides (AON) targeting the mRNA for the PDE4B/4D and 7A subtypes on lung inflammatory markers, both *in vitro *and *in vivo*.

**Methods:**

Normal human bronchial epithelial (NHBE) cells were transfected with FANA AON against PDE4B/4D and 7A alone or in combination. mRNA levels for target PDE subtypes, as well as secretion of pro-inflammatory chemokines were then measured following cell stimulation. Mice were treated with combined PDE4B/4D and 7A AON via endo-tracheal delivery, or with roflumilast via oral delivery, and exposed to cigarette smoke for one week. Target mRNA inhibition, as well as influx of inflammatory cells and mediators were measured in lung lavages. A two-week smoke exposure protocol was also used to test the longer term potency of PDE4B/4D and 7A AONs.

**Results:**

In NHBE cells, PDE4B/4D and 7A AONs dose-dependently and specifically inhibited expression of their respective target mRNA. When used in combination, PDE4B/4D and 7A AONs significantly abrogated the cytokine-induced secretion of IL-8 and MCP-1 to near baseline levels. In mice treated with combined PDE4B/4D and 7A AONs and exposed to cigarette smoke, significant protection against the smoke-induced recruitment of neutrophils and production of KC and pro-MMP-9 was obtained, which was correlated with inhibition of target mRNA in cells from lung lavages. In this model, PDE AONs exerted more potent and broader anti-inflammatory effects against smoke-induced lung inflammation than roflumilast. Moreover, the protective effect of PDE4B/4D and 7A AON was maintained when a once-weekly treatment schedule was used.

**Conclusion:**

These results indicate that inhaled AON against PDE4B/4D and 7A have unique effects on biomarkers that are believed to be important in the pathophysiology of COPD, which supports further development as a potential therapy in this disease.

## Background

Chronic obstructive pulmonary disease (COPD) is a complex syndrome characterized by chronic bronchitis, persistent cellular inflammation and progressive deterioration of airways and emphysema [1-3], for which cigarette smoking is by far the most important risk factor [4]. COPD is one of the leading causes of death and morbidity worldwide [5]. To date, no therapies have been shown to reduce mortality or prevent disease progression.

Although the composition of the lung cellular infiltrate varies among COPD patients, it is mainly constituted by neutrophils, macrophages and CD8^+ ^T cells [6]. The neutrophilic arm of airway inflammation is believed to be at the forefront of the lung pathogenesis in COPD patients [1,7-9]. In the airways, neutrophils can release a number of mediators including oxygen radicals, elastases and metalloproteases (MMP) that contribute to self-perpetuation of inflammation and promote matrix breakdown, leading to alveolar destruction and emphysema [10-12]. Patients with COPD have an increased number of neutrophils in broncho-alveolar lavages (BAL), sputum, airways and lung parenchyma [8,9], which directly correlates with disease severity [13]. Their recruitment and accumulation in the airways is driven by chemokines such as interleukin-8 (IL-8), the levels of which have been found to be increased in sputum, alveolar macrophages and bronchial epithelium obtained from COPD patients [14-16].

In airways, elevation of intracellular cAMP has been associated with the general suppression of activity of inflammatory cells and relaxation of airway and vascular smooth muscle [17]. Levels of intracellular cAMP are determined by the enzymatic balance between synthesis by adenylate cyclase and hydrolysis by phosphodiesterases (PDE). The PDEs represent a large family of isozymes [18], of which PDE4B and PDE4D isotypes are predominantly expressed in a variety of inflammatory and structural lung cells [17], and have been shown to modulate the inflammatory response [19,20]. Small molecule PDE4 inhibitors with broad spectrum anti-inflammatory effects have been shown to reduce inflammatory cell recruitment and improve lung function in animal models of COPD [21-23]. Orally active PDE4 inhibitors such as cilomilast and roflumilast have reached an advanced clinical stage [24-26]. However one major hurdle in their development has been overcoming the dose-limiting systemic side effects, of which headaches, nausea and vomiting are the most common manifestations [27]. Moreover, arteritis and vasculitis in the gastrointestinal tract and mesenteric blood vessels of rodents [28] and cardiac tissue of primates [29] have also raised a concern about their safety profile. Although delivery of PDE4 inhibitors via inhalation could represent an alternative approach [22,30], the efficacy and safety of inhaled small molecules PDE4 inhibitors remains to be demonstrated [31,32]. Consequently, improving the therapeutic ratio of PDE4 inhibitors still represents an important challenge.

One strategy to overcome these limitations is to develop more selective PDE4 inhibitors. For example, as systemic PDE4D inhibition appears to be responsible for the emetic effect [33], PDE4B-specific inhibitors might provide better therapeutic ratios. However, as PDE4D also has a predominant role in the activation of T cells [34], PDE4B-specific inhibitors might display reduced efficacy as compared to inhibitors targeting both isoforms in the lung. Another interesting approach would be to develop inhibitors of other PDE classes to be used in combination with PDE4 inhibitors, with the goal of maintaining or enhancing efficacy while reducing the dose-limiting side effects associated with these drugs [35]. In this regard, PDE7 might represent a good candidate, as it is widely expressed in lung tissue and inflammatory cells [36]. Moreover, in vitro studies have shown that the anti-inflammatory activity of the PDE4 inhibitor rolipram was enhanced by a PDE7 inhibitor, although the PDE7 inhibitor alone was without effect [37].

Antisense oligonucleotides (AON), by their ability to downregulate the expression of specific proteins, represent an innovative therapeutic strategy for lung diseases [38-40]. AONs can inhibit gene expression through formation of duplexes with complementary mRNA, activation of RNAseH and degradation of the duplexes or blockade of the translation machinery [41-43]. The lung is lined with surfactant, which is believed to facilitate AON uptake into cells following inhalation, without the need for specific carriers [44]. Moreover, inhaled AONs are metabolized mostly in the lung [45], therefore limiting systemic exposure [46]. Their clinical utility in lung diseases was illustrated in a recent study where inhaled AONs were used to inhibit the allergen-induced inflammation in asthmatic subjects [47]. We hypothesized that inhaled AONs against selected PDE subtypes, by downregulating the expression of their targets locally, would reduce inflammation associated with cigarette smoke exposure. We reasoned that a combination of AONs against PDE4 and PDE7 subtypes would mediate improved pharmacological activity as compared to PDE4 only inhibitors. This study therefore examined the *in vitro *and *in vivo *potency of a combination of phosphorothioate AONs directed against PDE4B/4D and PDE7A subtypes. These AONs incorporate 2'-deoxy-2'-Fluoro-β-D-Arabinonucleic Acid (FANA) modifications, which have been shown to provide increased potency and increased stability as compared to second generation phosphorothioate oligonucleotides [48,49].

## Methods

### Antisense oligonucleotides (AON)

AONs were obtained from the University of Calgary DNA Synthesis Laboratory (UCDNA) and were purified by anion-exchange HPLC. AON sequences were as follows (lowercase: DNA; bold uppercase: FANA; all with phosphorothioate linkages): human PDE4B/4D (5'-**TC**tgcccatgtct**CCCA**-3'); human PDE7A (5'-**TCAT**gagtggcagctgc**AATT**-3'); mouse PDE4B/4D (5'-**GG**ttgctcaggtctgc**ACAG**-3'); mouse PDE7A (5'-**TCCAG**atcgtga**GTGGC**-3'). Control sequences with mismatched nucleotides were as follows: for human PDE4B/4D (5'-**AG**acgggtacaga**GGGT**-3'); human PDE7A (5'-**AGTA**ctcaccgtcgacg**TTAA**-3'); mouse PDE4B/4D (5'-**GC**atggtctgcagtgg**ACTC**-3'); mouse PDE7A (5'-**TGCTC**aacgtct**GAGGG**-3').

### Cell culture and transfection

Normal human bronchial epithelial cells (NHBE) were cultured in antibiotic-free BEGM medium (Clontech) until 70–90% confluency was reached, and were then transfected for 24 h with the AONs using Lipofectamine 2000 (Invitrogen). Fresh media containing TNF-α, IL-1β and IFN-γ (10 ng/ml each; Peprotech) was added 4 h before the end of the transfection.

### Mouse models of cigarette smoke-induced lung inflammation. 1-week smoke model

The housing and care of mice (male C57Bl/6, Charles Rivers Laboratory) used in this study were provided according to protocols approved by Mispro Biotech's Institutional Animal Care and Use Committee, in conformity with Canadian Council on Animal Care (CCAC) guidelines. On days 1, 3, 6 and 8, groups of mice were anesthesized with ketamine and xylazine (60 mg/kg and 12 mg/kg, i.p.) and intubated. Aerosols of vehicle (endotoxin-free distilled water), AONs or controls were administered into the trachea using a microsprayer (Penn-Century). On days 6 to 9, other groups of mice were treated by gavage with vehicle (0.5% methylcellulose) or roflumilast (Rasayan). On days 6 to 9 (3 h after drug treatment), mice were nose-only exposed to the smoke of two 2R4f reference cigarettes (University of Kentucky) per day. Cigarette smoke was delivered to mice using a nose-only exposure system (Proto-Werx), at a rate of 1 puff (20 ml) per minute, as described previously [12,50]. ***2-week smoke model: ***On days 1, 2, 3, 4, 5, 8 and 15, groups of mice were treated with vehicle, AONs or controls as described above. On days 8 to 12 and 15 to 18, mice were nose-only exposed to the smoke of two 2R4f reference cigarettes (University of Kentucky) per day, as described above for the 1-week smoke model. Mice were killed by anesthetic overdose (sodium pentobarbital 88 mg/kg i.p.) and exsanguination 20 h following the last smoke exposure. Bronchoalveolar lavage (BAL) were performed as described previously [50]. Differential cell counts were determined on at least 300 cells using standard morphological criteria.

### mRNA quantification

Total RNA was extracted from NHBE cells using the RNeasy mini kit (Qiagen); cDNA was prepared with SuperScript™ II Reverse Transcriptase (Invitrogen) and combined d(T)^18 ^oligos (Biocorp) and PDE4B specific primers (TIB MOLBIOL). Quantitative real-time PCR was performed using the LC Fast Start SYBR Green reagent (Roche) and gene-specific primers (TIB MOLBIOL) (see Table 1) with the LightCycler2.0 instrument (Roche). PDEs mRNA expression levels in BAL cells were quantified using the Quantigene^® ^assay (Panomics). Gene expression was normalized to a reference gene (PPIB, GAPDH or β2 m as indicated).

**Table 1:** Real-time PCR primer sequences

Primer	Sequence 5'-3'	Annealing Temperature (°C)
huPDE4B for	tggcagacctgaagacaatg	59
huPDE4B rev.1	aaattcctccatgatgcgg	
huPDE4B rev.2^a^	tctttgtctccctgctgga	
		
huPDE4D for	cagaatatggtgcactgtgc	59
huPDE4D rev	agtctatgaagcccacctgtg	
		
huPDE7A for	tcaggccatgcactgttact	57
huPDE7A rev	cctgattctctcaataagccc	
		
huPbib for	agagcatctacggtgagcg	57
huPbib rev	cttccgcaccacctcca	

^a^Reverse primer used for first strand reaction

### Cytokines and chemokines quantification

ELISA kits for human IL-8 (BD Biosciences), human monocyte chemoattractant protein-1 (MCP-1; R&D Systems), and murine KC (R&D Systems) were used. Mouse pro-MMP-9 was quantified using the SearchLight array testing service (Thermo Fisher Scientific).

### Statistical analysis

Results are expressed as mean ± standard error to the mean (SEM). Statistical comparisons between groups were carried out using unpaired t-test.

## Results

### Target mRNA knock down by PDE 4B/4D and PDE 7A antisense oligonucleotides in human broncho-epithelial cells

AON sequences were designed to specifically target the mRNA for human PDE4B/4D and synthesized with a phosphorothioate backbone. The high sequence homology between the catalytic domains of PDE4B and PDE4D genes enabled the design of single AON sequences specific for both isotypes. Phosphorothioate AON sequences were also designed to specifically target the mRNA for human PDE7A. Following screening for inhibition of target mRNA expression in 293 cells and in A549 cell lines, lead AON sequences were characterized by dose-response and time course analysis. For the most potent AONs, chemistry optimisation was performed by including FANA substitutions in a gapmer configuration in order to improve efficacy and duration of action, as well as to improve resistance to nuclease degradation. From this screening, two AON sequences were selected for this study, and identified as AON 4B/4D and AON 7A. Base mismatched oligonucleotides were used as control sequences.

**Figure 1:**
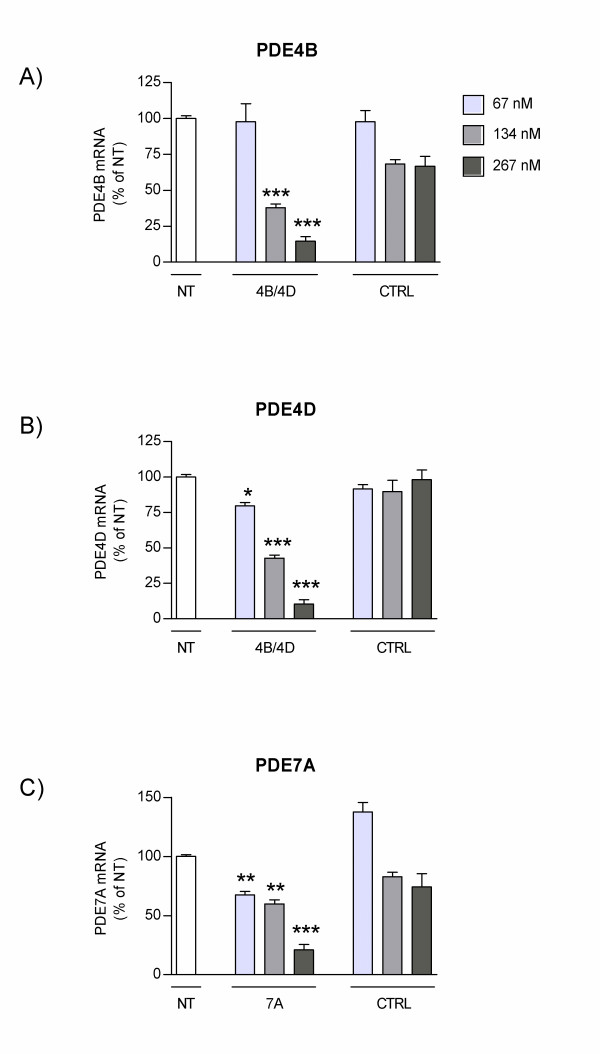
**Effect of 4B/4D and 7A antisense oligonucleotides (AON) on target mRNA expression in NHBE cells**. NHBE cells were transfected for 24 h with AON 4B/4D, with AON 7A, or with control sequences (CTRL) at 67, 134 or 267 nM, or were not transfected (NT). 4 h before the end of the transfection period, fresh media containing TNF-α, IL-1β and IFN-γ was added. mRNA levels for **(A) **PDE4B, **(B) **PDE4D and **(C) **PDE7A were quantified by real-time PCR analysis and normalized to a reference gene (Ppib). Results are expressed as percentage of PDE mRNA levels in non-transfected cells (± SEM). *: p < 0.05; **: p < 0.01, ***: p < 0.001, t-test relative to CTRL (n = 3 to 9 replicates).

As epithelial cells lining the airways are believed to play a critical role in the modulation of the inflammatory response in COPD, we first assessed the effect of AON 4B/4D and AON 7A on PDE expression levels in NHBE cells. NHBE cells were transfected with AON 4B/4D or AON 7A at three different doses, and PDE mRNA levels were determined 24 h later by real-time PCR analysis. Results indicate that AON 4B/4D reduced PDE4B (Figure 1A) and PDE4D (Figure 1B) mRNA in a dose-dependent manner. At the mid (134 nM) and the high (267 nM) doses, both PDE4B and PDE4D levels were significantly lower in cells transfected with the 4B/4D AON as compared to cells transfected with the control sequence. This provides evidence that PDE4B and 4D mRNA inhibition is sequence-specific and consistent with an antisense mechanism of action. Dose-dependent and sequence-specific down-regulation of PDE7A mRNA expression was also achieved following transfection with AON 7A (Figure 1C).

### PDE4B/4D and 7A mRNA knock down by antisense oligonucleotides correlates with inhibition of inflammatory mediators production by human broncho-epithelial cells *in vitro*

**Figure 2:**
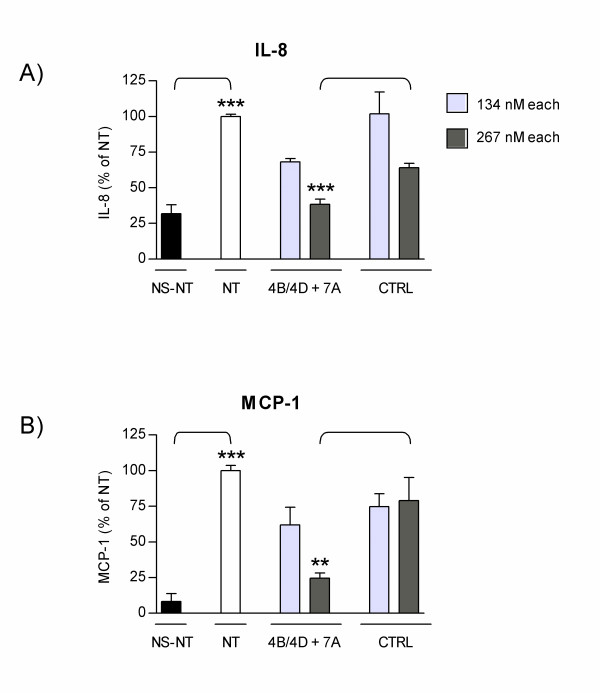
**Inhibition of inflammatory mediators by combined 4B/4D and 7A AONs in NHBE cells**. NHBE cells were transfected for 24 h with combined 4B/4D and 7A AON or with control sequences (CTRL) at 134 or 267 nM each, or were not transfected (NT). 4 h before the end of the transfection period, fresh media containing TNF-α, IL-1β and IFN-γ was added. Non-stimulated, non-transfected cells are shown as NS-NT. IL-8 **(A) **and MCP-1 **(B) **proteins were measured in cell culture supernatants by ELISA. Results are expressed as percentage of chemokine levels in non-transfected cells (± SEM). **: p < 0.01; ***: p < 0.001; t-test versus NS-NT or versus CTRL, as indicated (n = 6 replicates).

We next evaluated the effect of combined 4B/4D and 7A AONs on the functional activity of NHBE cells *in vitro*. Cells were transfected with a combination of 4B/4D and 7A AONs, each at 134 or 267 nM; cells were then stimulated and levels of IL-8 and MCP-1 produced were measured in cell culture supernatants by ELISA. Results indicate that the secretion of both IL-8 (Figure 2A) and MCP-1 (Figure 2B) were dose-dependently reduced by transfection with combined 4B/4D and 7A AONs. Levels of both chemokines were significantly lower in cells transfected with combined AONs at 267 nM as compared to controls, and were near baseline levels seen in non-stimulated, non-transfected cells. The strong inhibitory activity of combined 4B/4D and 7A AONs on IL-8 and MCP-1 secretion is in line with their ability to knock down their respective target mRNA (Figure 1).

### Efficacy of locally administered PDE4B/4D and PDE7A AON against cigarette smoke-induced lung inflammation in mice, and comparison with roflumilast

We next wanted to determine whether inhibition of PDE4B, PDE4D and PDE7A expression in the lung would protect against lung inflammation in mice exposed to cigarette smoke. Mouse specific AONs were designed, screened for target knock down in murine cell lines, and chemically modified to include FANA substitutions. One lead sequence specific for both mouse PDE4B and 4D mRNAs (AON 4B/4D), and one for mouse PDE7A (AON 7A) were selected, as well as control sequences, and used for all *in vivo *experiments.

**Figure 3:**
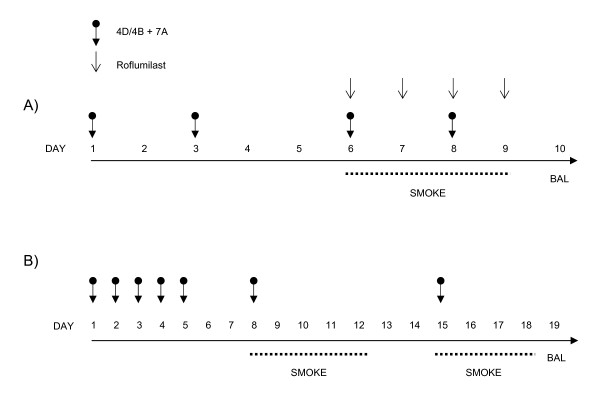
**Study protocols for cigarette smoke exposure in mice**. **A) **For the one-week smoke model protocol, mice were treated by endo-tracheal delivery of combined 4B/4D + 7A AON on days 1, 3, 6 and 8. On days 6 to 9, other groups of mice were treated daily with oral roflumilast. On days 6 to 9 (3 h after AON or roflumilast treatment), mice were nose-only exposed to the smoke of two 2R4f reference cigarettes per day. Bronchoalveolar lavages (BAL) were performed on Day 10. **B) **For the two-week smoke model protocol, mice were treated by endo-tracheal delivery of combined 4B/4D + 7A AON daily from Day 1 to Day 5, then once a week on Day 8 and 15. On days 8 to 12 and 15 to 18, mice were nose-only exposed to the smoke of two 2R4f reference cigarettes per day. BAL were performed on Day 19.

**Figure 4:**
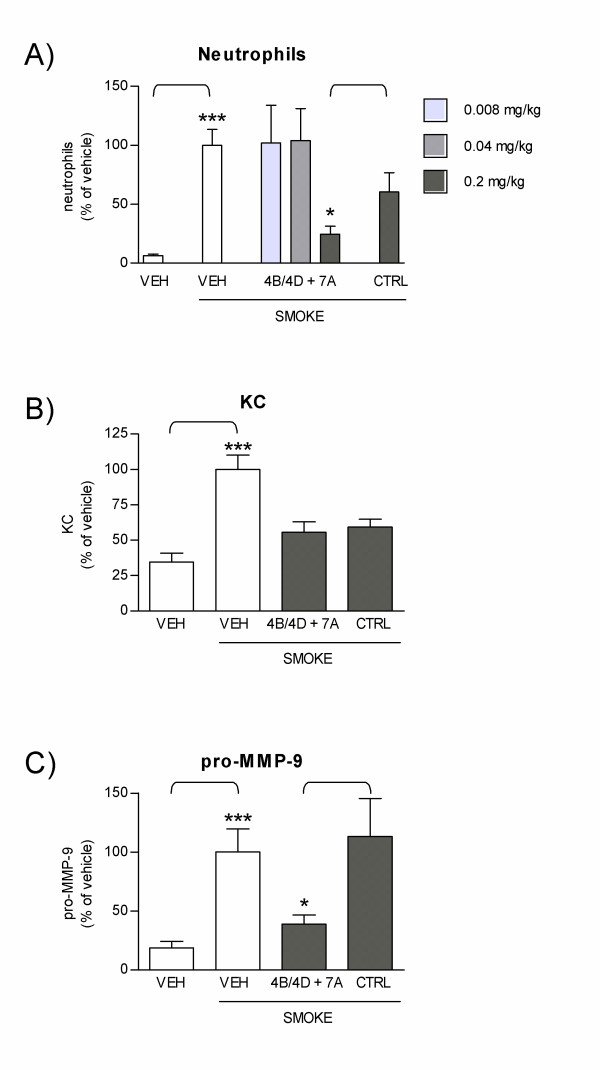
**Efficacy of treatment with combined 4B/4D and 7A AONs against cigarette smoke-induced lung inflammation**. Mice were treated with endo-tracheal vehicle only (VEH), with combined 4B/4D and 7A AON (0.008, 0.04 or 0.2 mg/kg/day) or with control sequences (CTRL, 0.2 mg/kg/day) and exposed to cigarette smoke for one week, as per protocol design described in Figure 3A. Neutrophil counts **(A)**, KC **(B) **and pro-MMP-9 levels **(C) **were measured in BAL. Results are expressed as percentage of neutrophils, KC or pro-MMP-9 levels in vehicle-treated and smoke-exposed animals (± SEM). *: p < 0.05; ***: p < 0.001; t-test versus vehicle-treated without smoke exposure, or versus CTRL, as indicated (compilation of 4 independent experiments; n = 10–20 mice per group for neutrophils, 14–15 per group for KC, and 9–10 per group for pro-MMP-9).

Sub-acute exposure of mice to cigarette smoke leads to lung inflammation which is comparable to the inflammatory response observed in COPD patients [21,51]. We used a nose-only sub-acute smoke model to assess the effect of local inhibition of PDE4B, PDE4D and PDE7A mRNA on neutrophilic inflammation. Mice were treated by endo-tracheal delivery of combined 4B/4D and 7A AON every other day, prior to and during cigarette smoke exposure, which lasted for one week (Figure 3A). Repeated smoke exposure induced a significant recruitment of neutrophils in broncho-alvoalar lavage (BAL) collected the day after the last smoke exposure (Figure 4A). Neutrophils counts in vehicle-treated and smoke exposed animal were 5.1 × 10^4 ^± 8.5 × 10^3 ^compared to 3.2 × 10^3 ^± 0.9 × 10^3 ^in mice treated with vehicle but not exposed to smoke. This was accompanied by increased secretion of KC (54.7 ± 10.5 pg/ml in smoke-exposed versus 17.5 ± 3.0 pg/ml in non-exposed), an important neutrophil chemoattractant considered to be the functional homolog for human IL-8 [52], and pro-MMP-9 (275.1 ± 91.2 pg/ml in smoke-exposed versus 72.1 ± 28.7 pg/ml in non-exposed), a metalloprotease produced by neutrophils and believed to play a role in lung tissue damage [10] (Figure 4B–C). When mice were treated with combined 4B/4D and 7A AON at 0.2 mg/kg/day, the smoke-induced neutrophil influx was significantly reduced when compared to mice treated with control sequences (59% inhibition, Figure 4A). At this dose of AONs, neutrophils, KC, and pro-MMP-9 all returned to near baseline levels seen in mice treated with vehicle only and not exposed to smoke (Figure 4A–C). Treatment of mice with lower doses of 4B/4D and 7A AON (0.008 and 0.04 mg/kg/day) was without effect. Although macrophages were also significantly increased in mice exposed to smoke (5.9 × 10^5 ^± 4.6 × 10^4 ^versus 3.4 × 10^5 ^± 2.9 × 10^4 ^in non-exposed mice), treatment with PDE AONs had no effect on macrophages. Lymphocytes were not significantly increased in this 1-week smoke model (data not shown).

**Figure 5:**
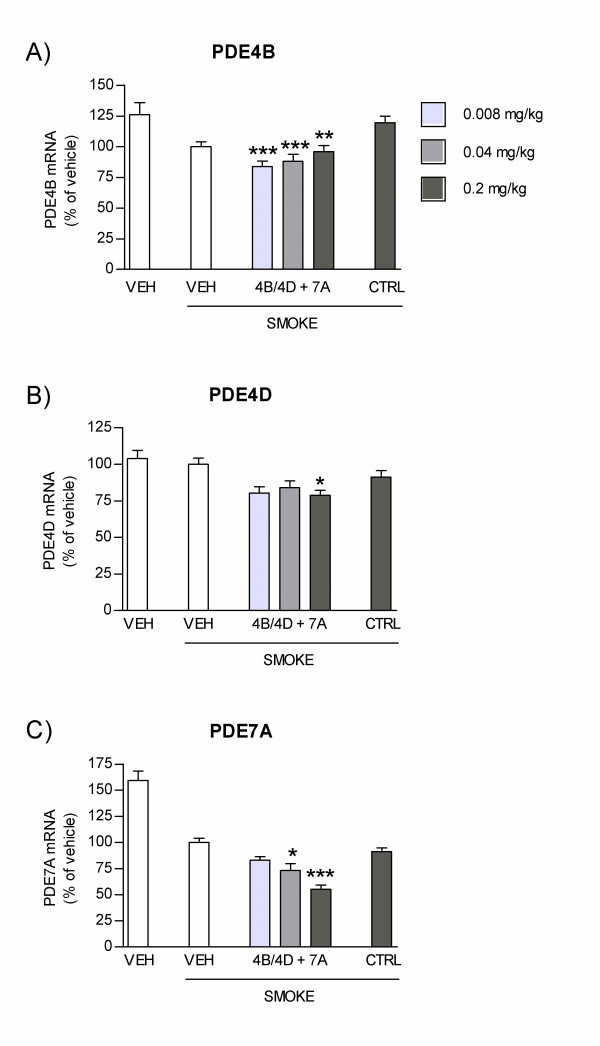
**Target mRNA expression in BAL cells following treatment with 4B/4D and 7A AON and smoke exposure**. Mice were treated with combined 4B/4D and 7A AON (0.008, 0.04 or 0.2 mg/kg/day) or with control sequences (CTRL, 0.2 mg/kg/day) and exposed to cigarette smoke for one week, as per protocol design described in Figure 3A. PDE4B **(A)**, PDE4D **(B) **and PDE7A **(C) **mRNA expression were quantified in BAL cells lysates using the Quantigene^® ^assay, and normalized to the expression of GAPDH used as a reference gene. Results are expressed as percentage of PDE mRNA levels in vehicle-treated and smoke-exposed animals (± SEM). *: p < 0.05; **: p < 0.01; ***: p < 0.001; t-test versus CTRL (compilation of 4 independent experiments, n = 10 to 20 mice per group).

To determine whether inhibition of neutrophil recruitment was correlated with PDE mRNA inhibition in vivo, the expression of target mRNAs was measured in BAL cells collected the day after the last smoke exposure. Results showed that for groups in which a significant reduction of neutrophils was obtained (treated with 0.2 mg/kg/day, Figure 4A), there was also a significant reduction of mRNA levels for all three PDE targets (0.2 mg/kg/day, Figure 5A–C).

**Figure 6:**
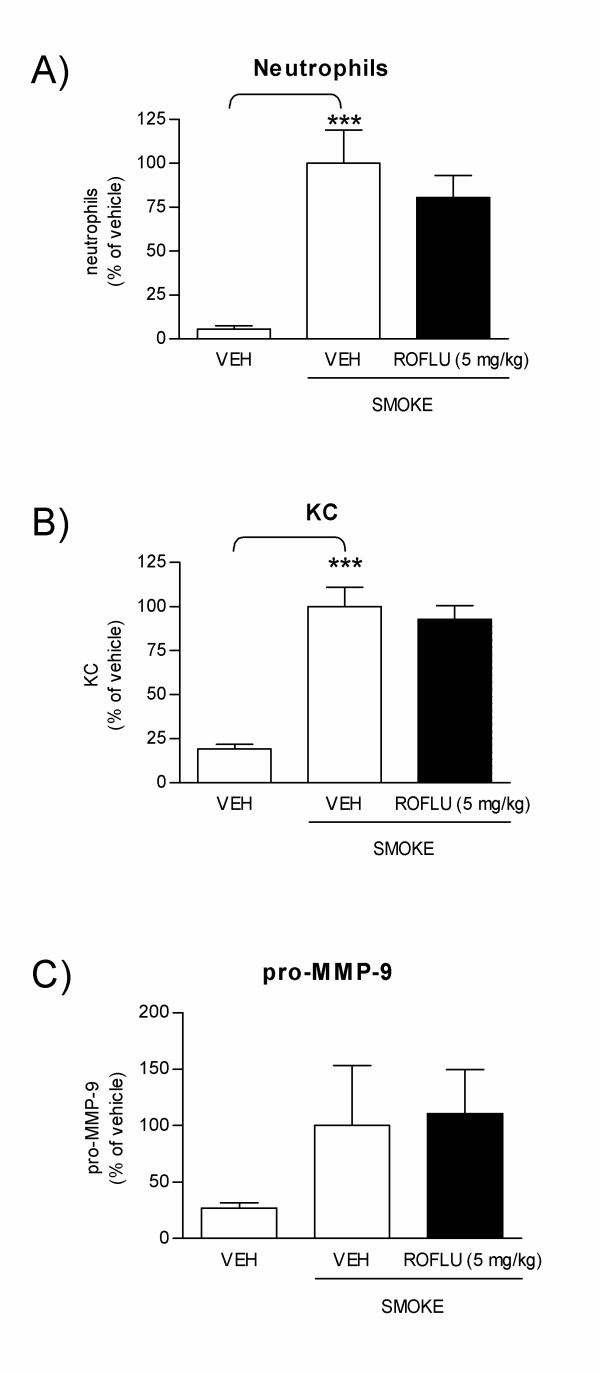
**Effect of treatment with roflumilast on cigarette smoke-induced lung inflammation**. Mice were treated with oral vehicle only or with oral roflumilast (5 mg/kg/day) and exposed to cigarette smoke for one week, as per protocol design described in Figure 3A. Neutrophil counts **(A)**, KC **(B) **and pro-MMP-9 levels **(C) **were measured in BAL. Results are expressed as percentage of neutrophils, KC or pro-MMP-9 levels in vehicle-treated and smoke-exposed animals (± SEM). ***: p < 0.001; t-test versus vehicle-treated without smoke exposure (compilation of 4 independent experiments, n = 12–21 mice per group for neutrophils and KC, and 3–6 per group for pro-MMP-9).

The potency of combined 4B/4D and 7A AON at reducing cigarette smoke-induced lung inflammation was compared to roflumilast. The route of administration (oral) and dosing regimen (5 mg/kg/day) used for roflumilast (Figure 3A) was based on a previous report showing inhibitory activity against neutrophils in mice acutely exposed to cigarette smoke [21]. In contrast to the effects observed with the 4B/4D and 7A AON, roflumilast had a small but non-significant effect on the smoke-induced neutrophil influx (Figure 6A) and did not reduce the secretion of KC or pro-MMP-9 (Figure 6B–C).

### Sustained anti-inflammatory activity of PDE4B/4D and PDE7A AON following once a week treatment in cigarette smoke-exposed mice

**Figure 7:**
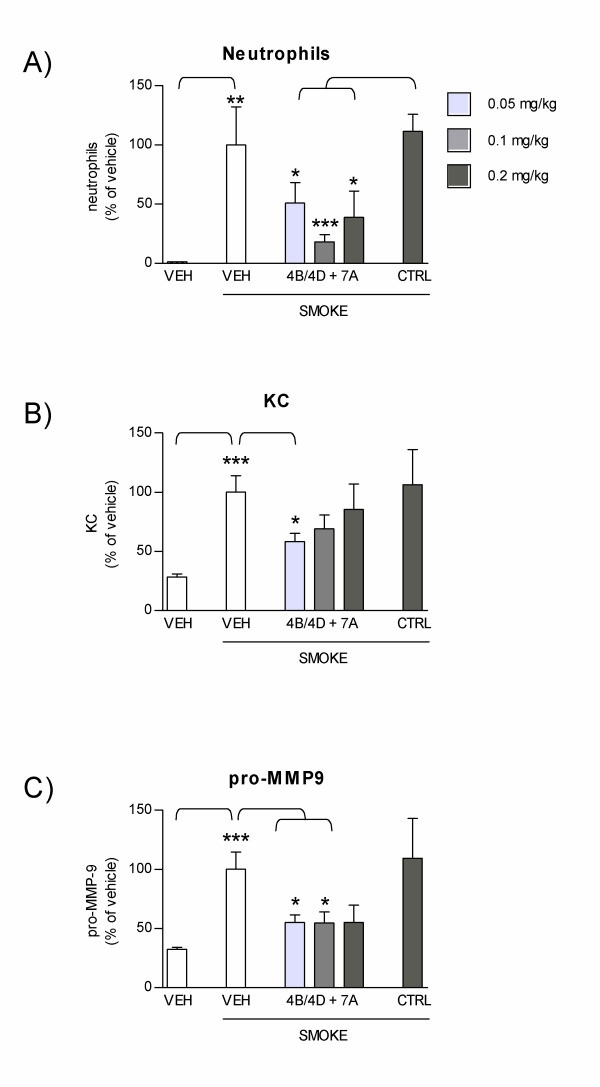
**Sustained effect of 4B/4D and 7A AONs following once a week treatment and two weeks of smoke exposure**. Mice were treated with endo-tracheal vehicle only (VEH), with combined 4B/4D and 7A AON (0.05, 0.1 or 0.2 mg/kg/day) or with control sequences (CTRL, 0.2 mg/kg/day) daily for one week, then once a week during the 2-week smoke exposure period, as per protocol design described in Figure 3B. Neutrophil counts **(A)**, KC **(B) **and pro-MMP-9 levels **(C) **were measured in BAL. Results are expressed as percentage of neutrophils, KC or pro-MMP-9 levels in vehicle-treated and smoke-exposed animals (± SEM). *: p < 0.05; **: p < 0.01; ***: p < 0.001; t-test versus vehicle-treated without smoke exposure, versus vehicle-treated or versus CTRL, as indicated (n = 4 to 8 mice per group).

The longer term efficacy of 4B/4D and 7A AON administered to the lungs was then evaluated in a two-week smoke exposure protocol, in which mice were treated daily with combined AONs for one week prior to smoke exposure, then once a week during the period of smoke exposure (see Figure 3B). As for the one week protocol shown above, repeated smoke exposure for two weeks resulted in a significant recruitment of neutrophils (1.0 × 10^5 ^± 3.2 × 10^4 ^cells) and production of KC (78.7 ± 10.8 pg/ml) and pro-MMP-9 (268.8 ± 38.8 pg/ml) in the airways of mice (Figure 7A–C). In mice treated with combined 4B/4D and 7A AONs, neutrophil recruitment was significantly blocked as compared to control mice, with doses of AONs as low as 0.05 mg/kg/day (54% inhibition, Figure 7A). The secretion of KC and pro-MMP-9 were reduced by combined AON treatment in a similar fashion (Figure 7B and 7C). These results indicate that protection against the smoke-induced recruitment of neutrophils and release of KC and pro-MMP-9 is still effective four days after the last treatment with combined PDE AONs. As for the 1-week smoke model, macrophages were significantly increased in mice exposed to smoke for two weeks (5.5 × 10^5 ^± 4.2 × 10^4^), but were not affected by PDE AON treatment. On the other hand, lymphocytes were increased following two weeks of smoke exposure (2.7 × 10^4 ^± 5.7 × 10^3 ^versus 5.5 × 10^3 ^± 1.2 × 10^3 ^in non-exposed mice), and were significantly inhibited by PDE AON treatment (66% inhibition versus CTRL at 0.1 mg/kg, p < 0.05, data not shown).

## Discussion

Much interest in the field of COPD has focused on strategies aimed at reducing the underlying inflammation through broad inhibition of the PDE4 isoforms. Orally administered second generation PDE4 inhibitors, such as cilomilast and roflumilast, have undergone extensive investigation and are currently in Phase III clinical development. Although effective, dose-limiting side effects related to the systemic exposure of these drugs have hampered their clinical development. Therefore, there is still a need for more selective and/or more potent PDE inhibitors with improved therapeutic ratios.

Increased interest in the therapeutic use of AONs has been fueled by recent pre-clinical [38,44,53-55] and clinical studies [47] showing their potential benefit in respiratory disorders involving inflammation such as asthma. We have developed FANA-modified antisenses oligonucleotides specifically targeting PDE isotypes 4B, 4D and 7A, as a potential new inhaled drug for the treatment of COPD. In NHBE cells, high levels of PDE4B, 4D and 7A mRNA inhibition were correlated with reduced functional activity, as illustrated by dose-dependent inhibition of IL-8 and MCP-1 secretion. The potency of combined PDE4B/4D and 7A AON on inflammatory markers in vitro is in line with their protective effect in vivo, as evidenced by their ability to reduce the smoke-induced recruitment of neutrophils and secretion of KC and MMP-9 in mice. Inhibition of neutrophil recruitment was also associated with PDE mRNA inhibition in vivo. Interestingly, we observed that the dose required to achieve significant inhibition of the three PDE targets at the mRNA level (0.2 mg/kg/day) was the same as the dose required to block the recruitment of neutrophils, KC and pro-MMP-9. At lower doses where only PDE4B mRNA inhibition was measured, no reduction in neutrophil recruitment was observed (see Figures 4A and 5A). Because PDE4B, 4D and 7A are expressed to different levels in different cell types, these data do not allow us to determine in which cellular subset in the BAL fluid the inhibition of PDE mRNA was the most efficient. The fact that total RNA from the mixed cell populations present in the BAL was measured might reflect this limitation. Nevertheless, these results are consistent with the idea that downregulating different PDE subtypes in different cell populations which together orchestrate the inflammatory response is required to achieve a protective effect against neutrophils in vivo. Moreover, and as previously shown in PDE4B and PDE4D knock out mice [20], these data support the idea that different PDE isotypes play complementary roles in the control of inflammatory cell recruitment. In the acute smoke exposure protocol used here (1-week model), orally delivered roflumilast at 5 mg/kg/day partially reduced neutrophil recruitment (20% reduction), but statistical significance could not be reached, and had no effect on KC or pro-MMP-9 secretion (Figure 6). Martorana *et al*. reported that oral roflumilast at 5 mg/kg inhibited neutrophils by 30% following one acute (20 minutes) exposure to cigarette smoke [21]. The longer smoke exposure period used here might explain the difference between the studies. Yet, the greater efficacy of the combined AONs PDE4B/4D and PDE7A supports the hypothesis that the anti-inflammatory activity of PDE4 inhibitors might benefit from the addition of a PDE7 inhibitor.

Besides neutrophils, lung macrophages are believed to play an important role in COPD, in part by their ability to release TNF-α, which can drive neutrophil influx and activate MMP-12 release, leading to extracellular matrix degradation [50]. In both the 1-week and the 2-week smoke models used here, macrophages were significantly increased in lung lavages upon smoke exposure. However, no reduction in macrophage counts were observed in mice treated with PDE4B/4D and 7A AONs, nor with roflumilast (data not shown). Although roflumilast has been shown to reduce macrophage density in lung tissue of mice chronically exposed to smoke (7 months), no effect of roflumilast on macrophages was seen following acute smoke exposure in that study [21], which is in line with our results. Thus, lack of efficacy on macrophages in the acute smoke exposure protocols used here may reflect the limitations of the acute model, and therefore may not be predictive of the outcome in chronic smoke exposure regimens.

When tested in a two-week smoke exposure protocol, combined PDE4B/4D and 7A AONs (at 0.05 mg/kg/day) were found to significantly reduce neutrophil influx, as well as KC and pro-MMP-9 in lung lavages collected four days after the last AONs treatment (Figure 7). In addition, treatment with combined PDE4B/4D and 7A AONs also resulted in significant inhibition of lymphocyte recruitment (data not shown). Results from these experiments suggest that once a steady state level of AONs is reached in the lung, a once-a-week treatment regimen could be sufficient to keep cellular inflammation to low levels. Although the results from this 2-week smoke exposure protocol are encouraging, more studies are needed to verify whether protection against inflammation is maintained in more chronic smoke exposure models, and whether PDE AON treatment can be beneficial on long term endpoints such as emphysema.

Delivery of AON directly to the lung via inhalation presents key advantages over systemic delivery of small molecule PDE4 inhibitors. Inhalation of AONs achieves appreciable local lung concentrations at the site of action, at lower administered doses. In a 14-day inhalation study in monkeys, PDE-targeting FANA AONs were found to be safe and well tolerated at all dose levels tested (from 0.05 to 2.5 mg/kg/day) [46]. Moreover, pharmacokinetic studies indicated very low levels of AON in plasma (<1%), and no plasma accumulation was obtained after repeated doses. In humans, there were no detectable levels of AON in plasma following inhalation for four consecutive days of 1.5 mg of TPI ASM8, an AON drug candidate in development for the treatment of asthma [47].

## Conclusion

Findings from the present study show for the first time that a multi-target AON-based approach directed against specific PDE isoforms is effective at reducing key inflammatory markers characterizing COPD. While others have begun to explore the concept that combined or dual PDE4/PDE7 inhibitors may provide a better therapeutic ratio [35], our data support this idea and suggest that such an approach is valuable for potent inhibition of key inflammatory markers. Together, the potential favorable safety profile of locally delivered AON, combined with the improved efficacy resulting from direct access to target cells in the lung, support further development of PDE AON for chronic respiratory indications such as COPD and asthma.

## Competing interests

MF, HD, MEH, KM, SS, RS, PMR and NF are employed by Topigen Pharmaceuticals inc. JG, PA, SM and LP are former employees of Topigen Pharmaceuticals inc. PMR is founder of Topigen, invested 2,200,000.00$ in the company and currently owns approximately 1–2% of company stock. He has many patents received or pending but does not own any royalties. PMR has received a 27,000.00$ grant from Topigen to perform research on RSV.

## Authors' contributions

MF and HD participated in the conception, designed the studies, participated in their coordination, analysed the data and drafted the manuscript. MEH, PA, KM, SM and SS carried out in vivo drug treatment and smoke exposure, differential cell counts, mRNA quantification and immunoassays. JG performed in vitro transfections, mRNA quantification and immunoassays. RS, PMR and LP participated in the conception, design and coordination of the studies and critically reviewed the manuscript. NF conceived the studies, participated in their design and coordination, assisted in drafting and critically reviewed the manuscript.
